# Supplementary figures and images for: OperonSEQer: A set of machine-learning algorithms with threshold voting for detection of operon pairs using short-read RNA-sequencing data
Source: PLoS Comput Biol. 2022 Jan 5;18(1):e1009731. doi: 10.1371/journal.pcbi.1009731 (PMC8765615; doi:10.1371/journal.pcbi.1009731)

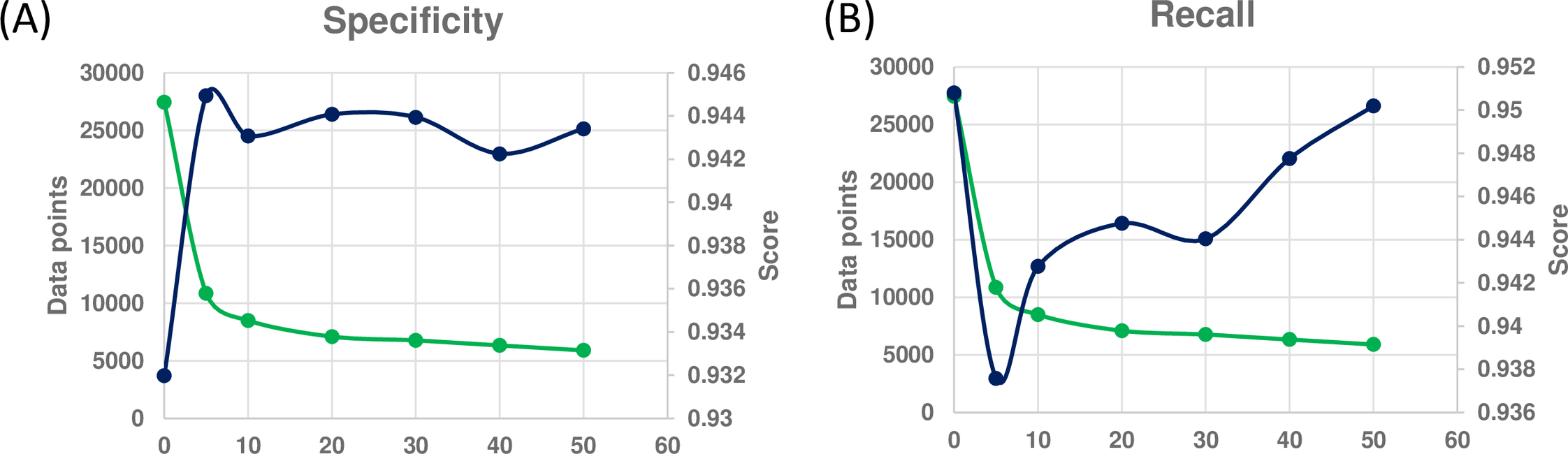

Supplement: S1 Fig — Sensitivity (A) and recall (B) tradeoff curves are shown, with the X-axis representing the mean coverage cutoff, the left Y-axis representing the number of data points retained as a result of the cutoff, and the right Y-axis representing the score. We determined that 10bp was a good cutoff based on the tradeoff between recall, specificity and number of data points. (TIF) [file pcbi.1009731.s001.tif]

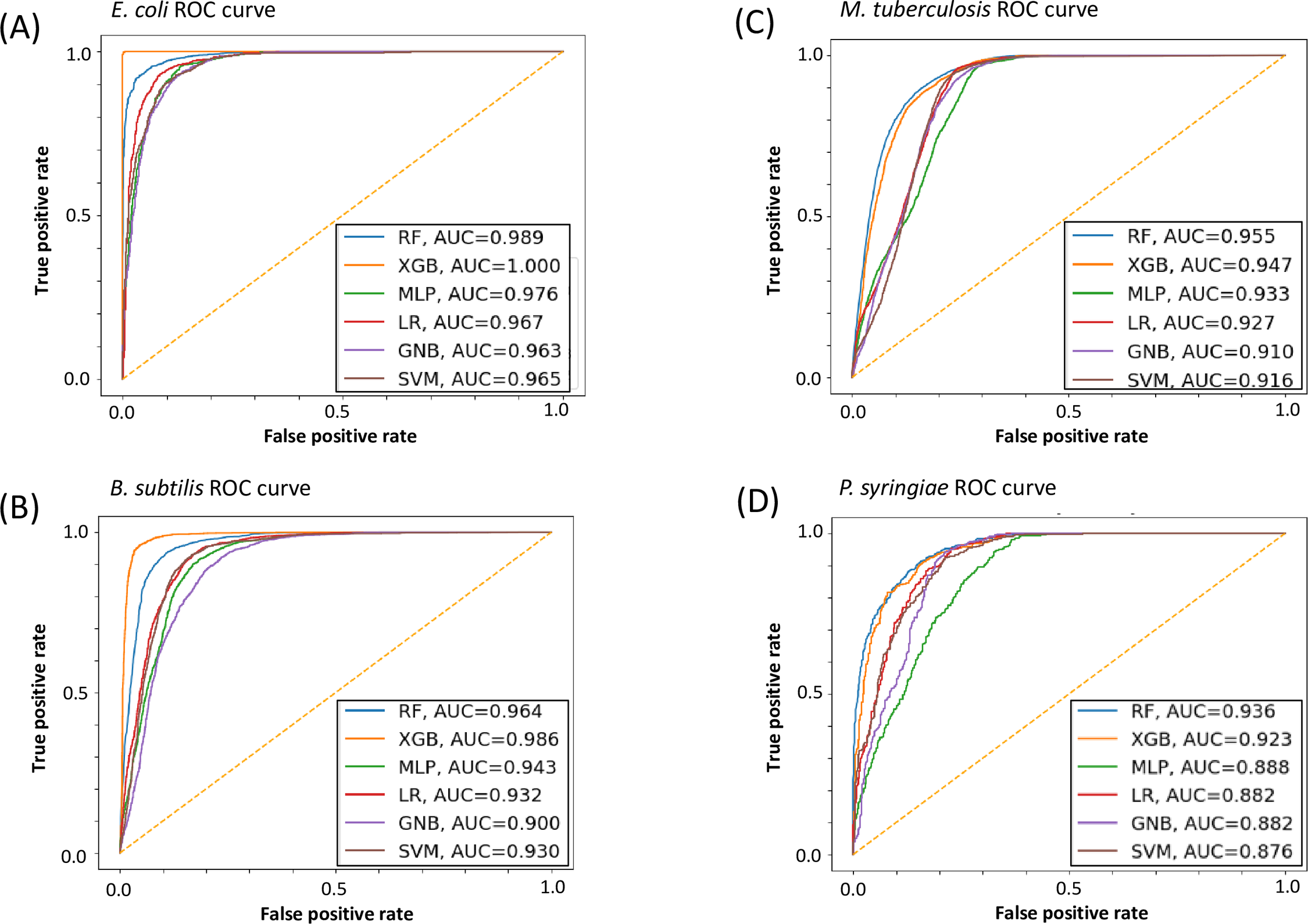

Supplement: S2 Fig — ROC (receiver operating characteristics) curves, and AUC (area under the curve) for the 7 algorithms in Operon-SEQer for the (A) E. coli, (B) B. subtilis, (C) M. tuberculosis, and (D) P. syringiae data sets. (TIF) [file pcbi.1009731.s002.tif]

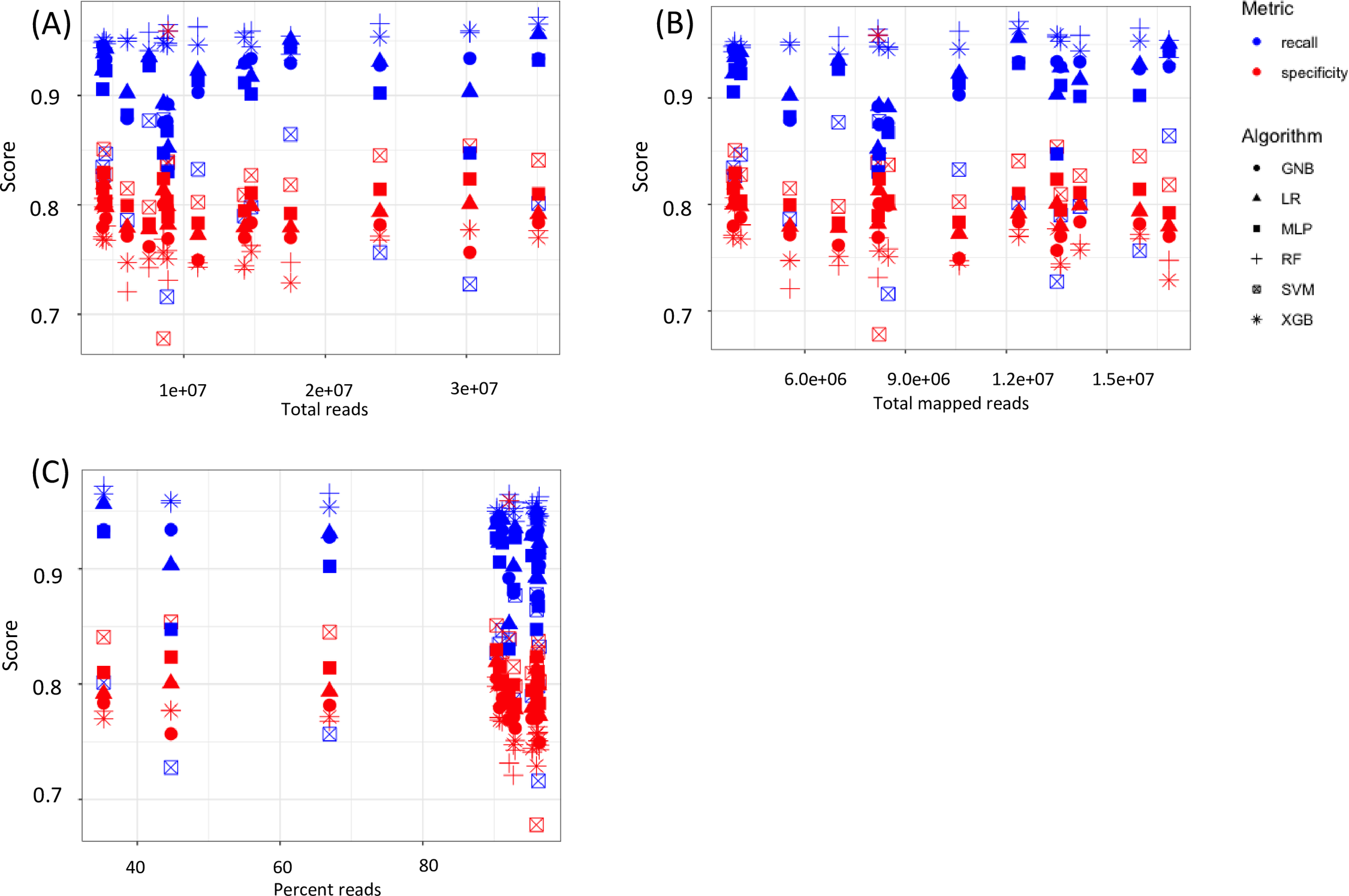

Supplement: S3 Fig — Relationship between recall (blue) and specificity (red) of the 6 algorithms of Operon-SEQer for (A) total reads, (B) total mapped reads, and (C) percent mapped reads in each data set from M. tuberculosis (PRJNA521480). (TIF) [file pcbi.1009731.s003.tif]

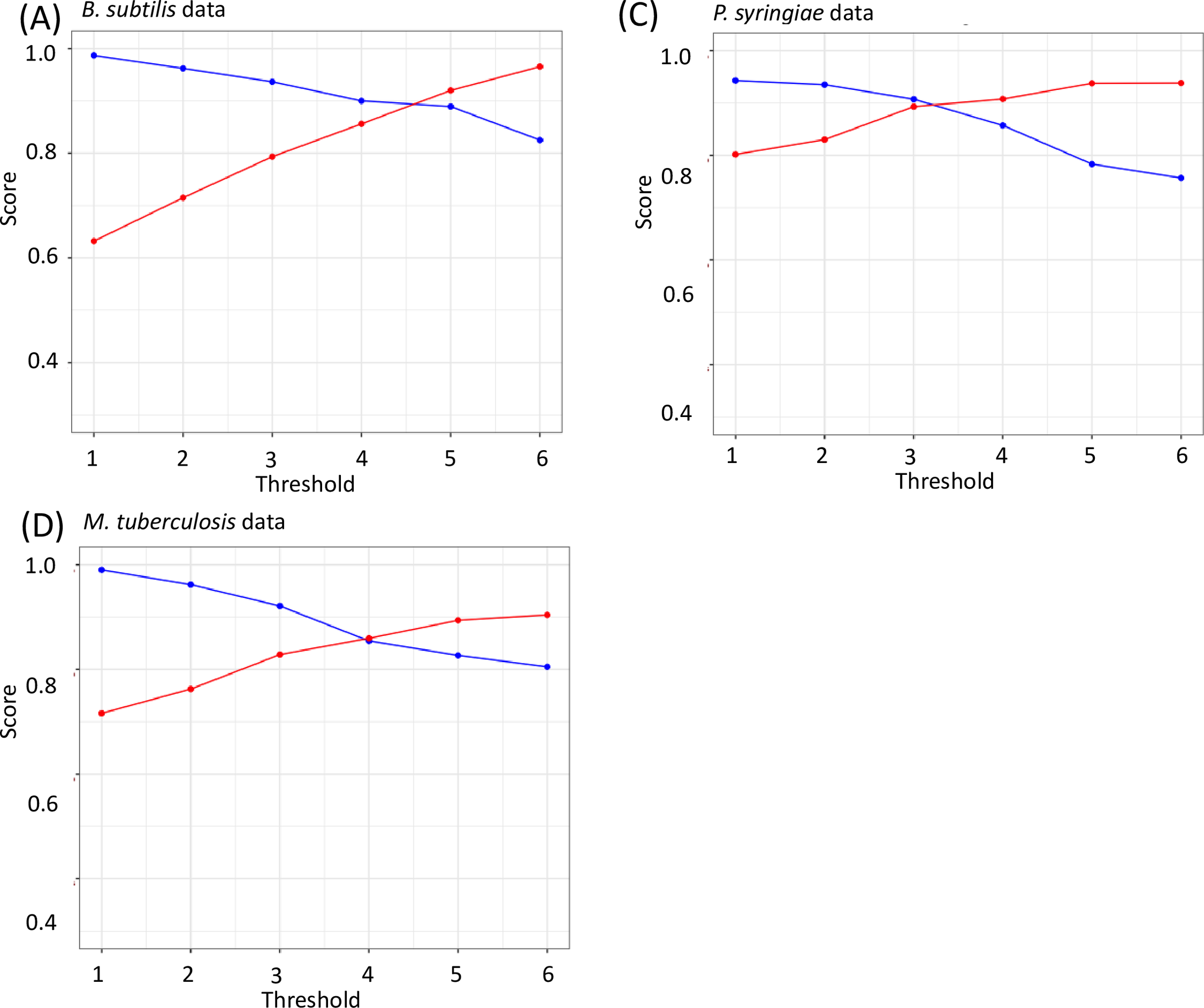

Supplement: S4 Fig — Recall (blue) and specificity (red) of the Operon-SEQer ensemble with algorithm agreement cutoffs of 1–6 for operon pair calls for the new data set from (A) B. subtilis, (B) P. syringiae, and (C) M. tuberculosis. (TIF) [file pcbi.1009731.s004.tif]

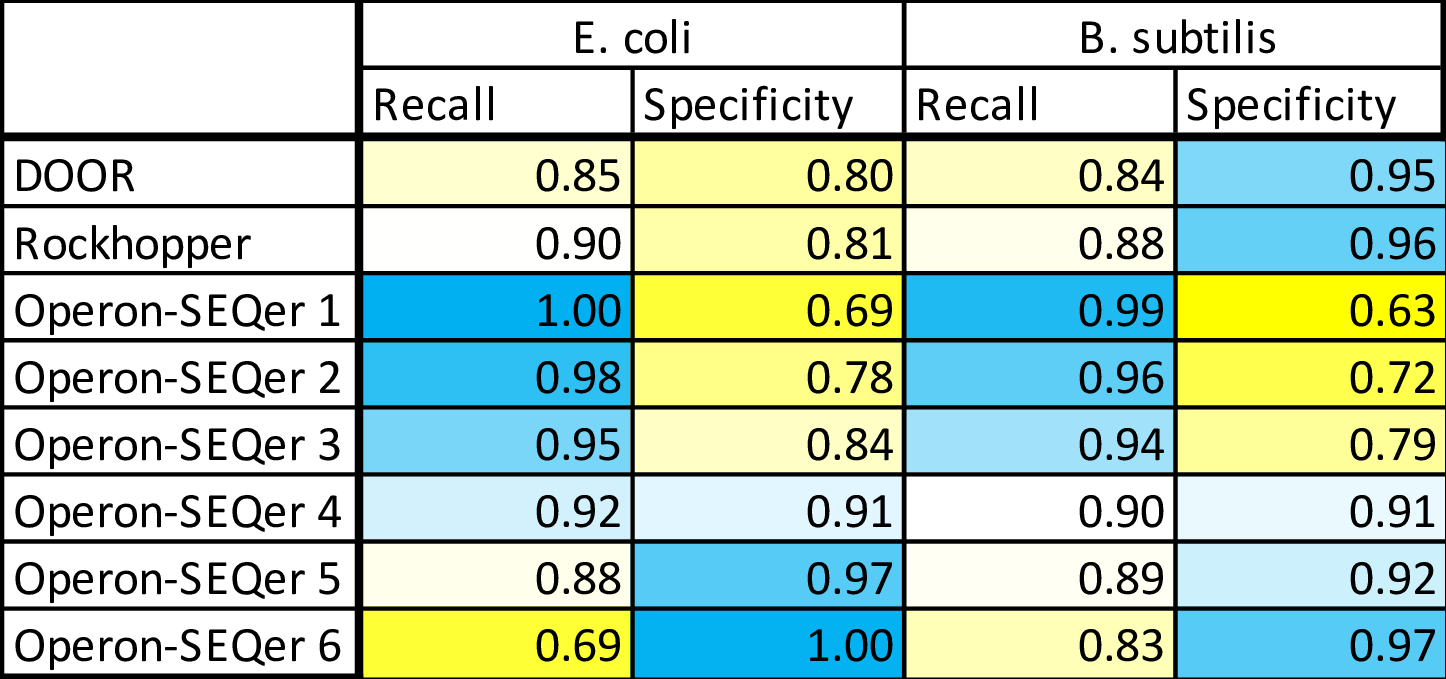

Supplement: S1 Table — Comparing the recall and specificity of DOOR and Rockhopper with the OperonSEQer ensemble (with agreement of anywhere between 1 and 6 of the algorithms that make up OperonSEQer being used to make operon pair calls). Heat map colors range from yellow (lowest) to white (mid-point) to blue (highest). (TIF) [file pcbi.1009731.s005.tif]

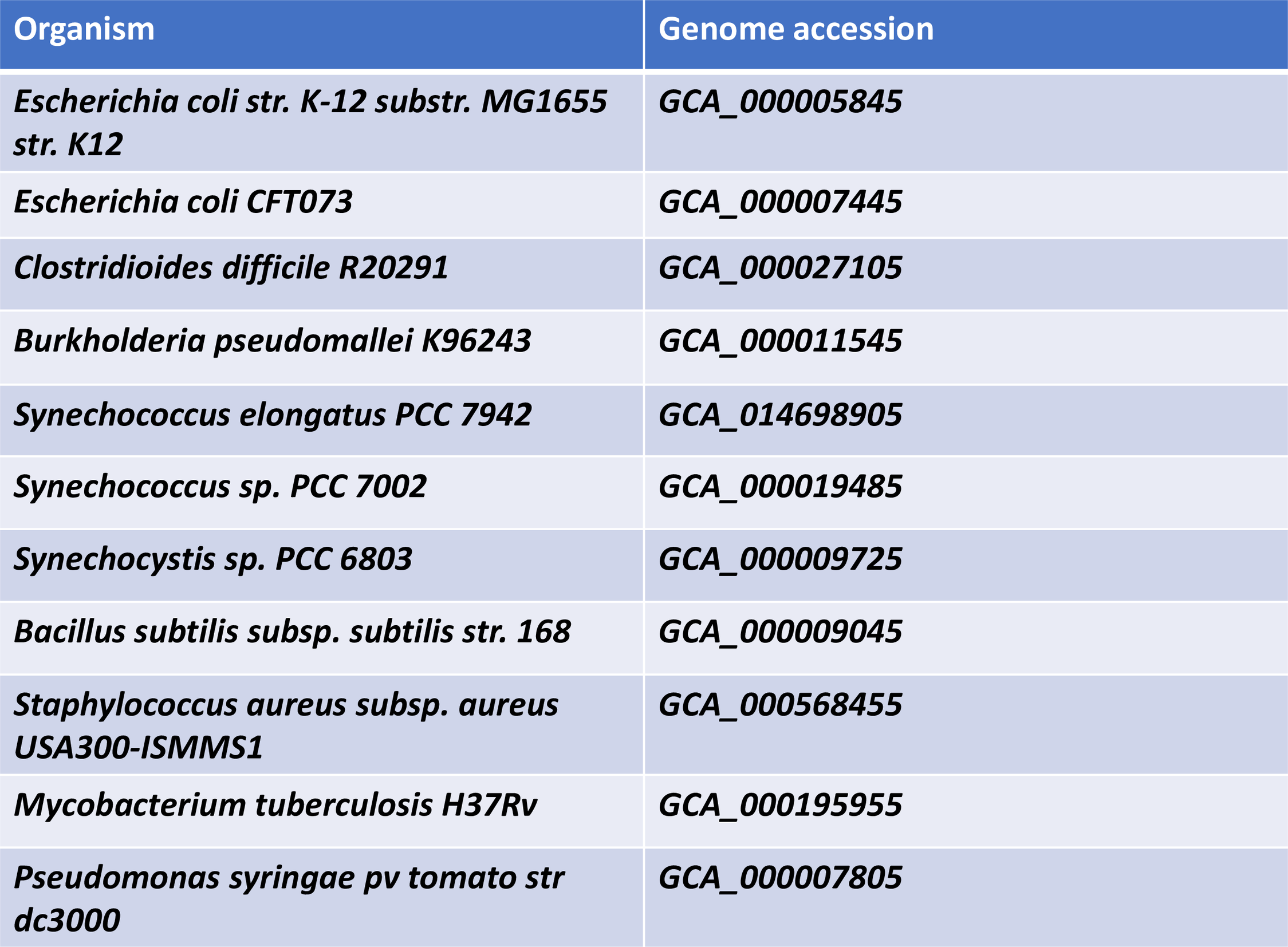

Supplement: S2 Table — (TIF) [file pcbi.1009731.s006.tif]
