# Supplementary material for: OperonSEQer: A set of machine-learning algorithms with threshold voting for detection of operon pairs using short-read RNA-sequencing data
Source: PLoS Comput Biol. 2022 Jan 5;18(1):e1009731. doi: 10.1371/journal.pcbi.1009731 (PMC8765615; doi:10.1371/journal.pcbi.1009731)
Supplement: Attachment — Submitted filename: review_operonSEQer.pdf [file pcbi.1009731.s007.pdf]

In this paper, the authors presented OperonSEQer, a new tool for *de-novo* operon prediction in prokaryotic genomes using RNA-seq data. Coverage values of gene pairs and their intergenic regions are compared through non-parametric analysis of variance. Then the test metrics are used as input in a wide set of machine learning (ML) models thus ensuring a flexible approach, not considering species, genes nor function. This approach was trained, tested and validated on a significant set of diverse bacterial species for which RNA-seq data was available. Interestingly, the use of a set of predictions from different ML models allowed the authors to implement a "threshold voting system" based on the agreement between models and offering the opportunity for users to choose a setup favoring high recall or high precision based on agreement between models.

Moreover, the authors had a relevant critical view on the data they used, especially the database of operons used as truth in the training of the models and the evaluation part. By comparing this database with their results as well as with SMRTseq transcriptomics data (long read transcriptomics) they were able to confirm that OperonSEQer can identify previously unidentified operons.

Overall, I think this paper can be of interesting value for the bacterial genomics field. First, the application of the software could be of high impact for novel operon detection and a better understanding of bacterial genomes organisation and function. The multi-model approach provides an interesting and flexible way of analyzing and discussing the results. Finally, the relevant use of publicly available data on top of the code available on a public GitHub repository ensures the reproducibility and transparency of the work.

However, the manuscript has some clarity and precision issues, especially regarding the non-parametric analysis of variance part and the assessment of novel operon detection using SMRTseq data. Indeed, some parts of the main text can remain unclear and could be easily improved by adding more details.

Major issues

Analysis of variance (Kruskal-Wallis test) : The features used in the ML models are described as the statistic and p-value of 3-way Kruskal-Wallis test (coverages of gene A, B and their intergenic region) as well as each 2-way test. In that case post-hoc pairwise tests might be more appropriate than 2-way KW tests as seen in the code (maybe Dunn's test instead). Moreover, multiple testing correction is not mentioned in the manuscript and should be needed for such analysis.

Mapping RNA-seq read on genome : As explained in the Method part, RNA-seq data are aligned across genomes and filter for genes only with a gff3 file retrieved from Ensembl Bacteria. As one could expect intra-species differences in terms of genome composition and operon presence/absence, the mention of the genomes used as reference for this analysis are absolutely needed since results will be impacted by the choice of a (set of) reference genome.

Prediction of new operon : Clarity issue in the paragraph ranging from line 235 to 259 in the manuscript as well as the Fig 4.A that is described in here. It is difficult to understand what the "fraction" represents. Especially in the way data should be interpreted. It is ">80%

of the operon calls made by Operon-SEQer are confirmed by standard and SMRT-seq data " while the manuscript (l. 247-248) seem to claim that when standard and SMRT seq agree, >80% of these calls can be found by Operon-SEQer. Same remarks for the end of the paragraph (l. 250-254).
In a nutshell, the clarification between "*X% of operons confirmed by Y are found by OperonSEQer*" and "*X% or the operons found by OperonSEQer are confirmed by Y*" would improve the explanation of this analysis and help support the proof of new operons discovery.
It would also be interesting and help the understanding to have absolute values available here on top of relative ones.

Minor issues

in Author Summary

- *l. 41-42* : distinction between the operon (the organization of the genetic information) and the polycistronic RNA (the molecule conveying it)

in Results

- *Results l. 156* : What's correlating is the likelihood (or another metric) of a pair of genes being in an operon and not the pair of genes.

- *l. 193* Figure 2b is mentioned but lacking in the provided manuscript
- *l. 214* Very strange transition between sentences that seem to talk about different issues
- *l. 226-227* Heatmap is mentioned as Supp. Fig. 3 while being Supp. Table 1 in the provided manuscript
- The difference between Figures 4C and 4D could be highlighted more, on the figure, the Title changes from "E. coli data" to "E. coli data with thresholds voting" while both figures display the impact of threshold voting.

In Discussion

- *l. 305* Missing reference ?

in Material and Methods

- *l. 415* Table in the main text without title or number

Misc

- References 21 to 37 not included in the main text
