# Supplementary material for: OperonSEQer: A set of machine-learning algorithms with threshold voting for detection of operon pairs using short-read RNA-sequencing data
Source: PLoS Comput Biol. 2022 Jan 5;18(1):e1009731. doi: 10.1371/journal.pcbi.1009731 (PMC8765615; doi:10.1371/journal.pcbi.1009731)
Supplement: Attachment — Submitted filename: OperonSEQer_ResponseToReviewers.docx [file pcbi.1009731.s008.docx]

OperonSEQer: A set of machine-learning algorithms with threshold voting for detection of operon pairs using short-read RNA-sequencing data

Response to Reviewer's Comments:

**Authors’ responses are in bold. Major changes to the text are highlighted in the manuscript.**

Reviewer #1: 

In this paper, the authors presented OperonSEQer, a new tool for de-novo operon prediction in prokaryotic genomes using RNA-seq data. Coverage values of gene pairs and their intergenic regions are compared through non-parametric analysis of variance. Then the test metrics are used as input in a wide set of machine learning (ML) models thus ensuring a flexible approach, not considering species, genes nor function. This approach was trained, tested and validated on a significant set of diverse bacterial species for which RNA-seq data was available. Interestingly, the use of a set of predictions from different ML models allowed the authors to implement a “threshold voting system” based on the agreement between models and offering the opportunity for users to choose a setup favoring high recall or high precision based on agreement between models.
Moreover, the authors had a relevant critical view on the data they used, especially the database of operons used as truth in the training of the models and the evaluation part. By comparing this database with their results as well as with SMRTseq transcriptomics data (long read transcriptomics) they were able to confirm that OperonSEQer can identify previously unidentified operons.
Overall, I think this paper can be of interesting value for the bacterial genomics field. First, the application of the software could be of high impact for novel operon detection and a better understanding of bacterial genomes organisation and function. The multi-model approach provides an interesting and flexible way of analyzing and discussing the results. Finally, the relevant use of publicly available data on top of the code available on a public GitHub repository ensures the reproducibility and transparency of the work.
However, the manuscript has some clarity and precision issues, especially regarding the non-parametric analysis of variance part and the assessment of novel operon detection using SMRTseq data. Indeed, some parts of the main text can remain unclear and could be easily improved by adding more details.

**Response: We thank the reviewer for their thorough review, and we hope that we have addressed their specific concerns, as detailed below.**

---Major issues---
Analysis of variance (Kruskal-Wallis test) : The features used in the ML models are described as the statistic and p-value of 3-way Kruskal-Wallis test (coverages of gene A, B and their intergenic region ) as well as each 2-way test. In that case post-hoc pairwise tests might be more appropriate than 2-way KW tests as seen in the code (maybe Dunn’s test instead). Moreover, multiple testing correction is not mentioned in the manuscript and should be needed for such analysis.

**Response: We agree with this reviewer’s comment on correcting for multiple hypothesis testing, and have therefore used a Dunn’s post-hoc p-value for the Kruskal-Wallis test. We have re-trained all our models and re-generated all our figures using the newly trained models. This had a very small effect on the exact metrics by which we judged our models, and the overall results and conclusions of the manuscript remain the same.**

Mapping RNA-seq read on genome : As explained in the Method part, RNA-seq data are aligned across genomes and filter for genes only with a gff3 file retrieved from Ensembl Bacteria. As one could expect intra-species differences in terms of genome composition and operon presence/absence, the mention of the genomes used as reference for this analysis are absolutely needed since results will be impacted by the choice of a (set of) reference genome.

**Response: We have included the accession numbers of the reference genomes used in this study in Supplemental Table 2.**

Prediction of new operon : Clarity issue in the paragraph ranging from line 235 to 259 in the manuscript as well as the Fig 4.A that is described in here. It is difficult to understand what the “fraction” represents. Especially in the way data should be interpreted. It is “>80% of the operon calls made by Operon-SEQer are confirmed by standard and SMRT-seq data '' while the manuscript (l. 247-248) seem to claim that when standard and SMRT seq agree, >80% of these calls can be found by Operon-SEQer. Same remarks for the end of the paragraph (l. 250-254).
In a nutshell, the clarification between “X% of operons confirmed by Y are found by OperonSEQer” and “X% or the operons found by OperonSEQer are confirmed by Y” would improve the explanation of this analysis and help support the proof of new operons discovery.
It would also be interesting and help the understanding to have absolute values available here on top of relative ones.

**Response: We agree with the reviewer that Figure 4A was confusing and have replaced it with a clearer figure, which compares the AUC scores for OperonSEQer and MicrobesOnline (the gold standard) when calling SMRT-seq operons. We have also clarified the text as the reviewer suggested, and have included the absolute values in Sup. Figure 4A.**

---Minor issues---

in Author Summary
* l. 41-42 : distinction between the operon (the organization of the genetic information) and the polycistronic RNA (the molecule conveying it)

**Response: Thank you for pointing out this error in terminology. We have edited lines 37-40 in the ‘ChangesTracked’ version of the manuscript to read: “One major molecular difference between unicellular and multicellular organisms is the way they express genes – rather than making individual RNA molecules for each gene like multicellular organisms, prokaryotes express operons (i.e., a group of genes coding functionally related proteins) in contiguous polycistronic RNA molecules.”**

in Results
* Results l. 156 : What’s correlating is the likelihood (or another metric) of a pair of genes being in an operon and not the pair of genes.

**Response: We have corrected lines 163-164 in the ‘ChangesTracked’ version of the manuscript to read: “We performed a correlation analysis to determine the probability that a pair of genes (gene A and gene B with intermediate region I) is in an operon using a number of important features from Kruskal-Wallis (KW) analysis of the RNA-seq data (Figure 2).”**

* l. 193 Figure 2b is mentioned but lacking in the provided manuscript

**Response: We thank you for catching this typo. This reference has been corrected (Table 1).**

* l. 214 Very strange transition between sentences that seem to talk about different issues

**Response: We apologize for the confusion. These two sentences are, in fact, talking about the same issue (higher recall and lower specificity). We have modified the text in the ‘ChangesTracked’ version of the manuscript to hopefully clarify this. Lines 224-225 now read “Another possible explanation is that a bias in recall and specificity was introduced by variability in the depth and coverage of the sequencing data.”**

* l. 226-227 Heatmap is mentioned as Supp. Fig. 3 while being Supp. Table 1 in the provided manuscript

**Response: This typo has been corrected.**

* The difference between Figures 4C and 4D could be highlighted more, on the figure, the Title changes from “E. coli data” to “E. coli data with thresholds voting” while both figures display the impact of threshold voting.

**Response: The titles for figures 4C and 4D have been clarified to both include threshold voting, and with 4D to include replicate agreement.**

In Discussion
* l. 305 Missing reference ?

**Response: We have inserted the missing reference**

in Material and Methods
* l. 415 Table in the main text without title or number

**Response: This table was designated as Table 2. Additionally Table 1 was moved into the main text, as described in the author guidelines.**

Misc
* References 21 to 37 not included in the main text

**Response: Bibliography was updated to only contain referenced texts**

Reviewer #2: The manuscript by Raga Krishnakumar and Anne M. Ruffing describes a ensamble of machine learning algorithms "OperonSEQer" that use RNA-seq data to call operon-pairs.

My review will focus on the practical perspective as I'm not an expert in the Machine Learning models applied in the manuscript. I found the manuscript pleasing to read and liked the step-by-step reporting on features that made it easy to follow the authors reasoning.

**Response: We thank the reviewer for their comments; it is important that this manuscript is useful for experimentalists, not just experts in ML. We hope that we have addressed your specific concerns, as detailed below.**

Specific comments:
- It could be made more clear if the authors are looking at operon-pairs or operons in general (which can constitute more than 2 genes). Does the software automatically extract potential larger operons? I assume that the information would be readily available by looking up the operon pairs, but for non-expert users it might be a convinient feature to have.

**Response: We have included a feature in the software to build operons beyond just operon pairs, and this feature is included in the updated version of the software. We have also made mention of it in the text.**

- MicrobesOnline is used as the "gold standard". However, as a non-expert I would like a brief description of how MicrobesOnline have called those operons in the first place.

**Response: We have included a brief description in the text of how MicrobesOnline calculated their operons.**

- line24: "high coverage" of operons could be confused in this context. I guess what you mean is high sensitivity or high specificity?

**Response: We apologize for the confusion. Yes, we are referring to high sensitivity/recall or high specificity; we have edited the text to include these terms.**

- Line385: It's mentioned that 10 reads for at least one gene is used to avoid false calls? Why was 10 reads chosen? From the text it is also a little unclear to me if it is 10 reads, or an average coverage of 10 that it is used. It would be relatively easy to subset the data to simulate the impact of coverage on the calling of operon-pairs.

**Response: We chose a cutoff above zero since we did not want to make calls on genes that were not expressed. We have included an analysis of coverage in increments of 10 performed, and determined that 10 was the best tradeoff between recall, sensitivity and loss of data points (Sup Fig 1).**

- line 389: Why was coverage only collected in a single 50 bp window at 3', middle and 5'? Why not use the entire gene? Anecdotal I remember experiments where there was quite some bias in coverage between the 3' and 5' of genes. If it is really only 50 bp from each element that is used, then it should also be added to Figure 1 to explain how the method works.

**Response: We chose the ends of the genes to compare to the intergenic region rather than the whole gene because, as the reviewer alludes to and as is outlined in Figure 1, there can be some variability across a gene body, mostly caused by technical issues. We want to minimize the capture of these technical issues by looking at regions that are very close to each other, and should therefore be expected to have a similar expression pattern if they are on the same molecule. If, as the reviewer says, these two genes come from two different RNA molecules, then not only would there be biological differences, but any technical differences are likely to be more pronounced, since there is no chance of the two regions being on the same read. To clarify, we have added this to Figure 1, and explained it in the text associated with this figure. But we also acknowledge the issue of likelihood of occurrence on the same read/fragment as a contributor to some technical skewing (as pointed out by another reviewer too), and to minimize this, we now go 50bp into the body of the gene and use the next 50bp (as available based on the length of the gene) as our input.**

Figure 1: While I do like conceptual figures, I think it would be much more impactful to take 3 real examples from e.g. E. coli with real gene-names, lenghts and coverages.

**Response: A figure of real data examples made using IGV is now included in Figure 1.**

Figure 3: What does the confidence intervals show? As you argue the largest difference would be different conditions and experimental batch effects. Hence, I'm not sure what the confidence interval here is ment to show?

**Response: The confidence intervals are derived from a 100-fold bootstrap of subsets of the data sampling 10% at a time. We agree with the reviewer that this was not clearly explained and have now added this explanation to the text.**

Figure 4: I guess it was only Ecoli where SMRT data was available for? Could be written explicit in the figure text.
- "SMRT only" has low rate of confirmed calls -> was this due to low coverage of SMRT seq or very specific conditions?
- A little confusing with the naming of SMRT "no" and "yes". As I understand it SMRT-No is the MicrobesOnline call (called "stand" in figure 4?), while SMRT-Yes is MicrobesOnline+SMRT.

**Response: We agree with the reviewer that Figure 4A was confusing and (as mentioned above in response to Reviewer #1 as well) we have replaced it with a clearer figure, which compares the AUC scores for OperonSEQer and MicrobesOnline (the gold standard) when calling SMRT-seq operons. We have also clarified the text as the reviewer suggested, and have included the absolute values in Sup. Figure 4A.**

Reviewer #3: In this manuscript, Raga Krishnakumar and Anne M. Ruffing describe a new approach to predict, from a single RNA-Seq data set, pairs of adjacent genes that are co-transcribed (i.e. within the same operon). The proposed approach, OperonSEQer, uses different “machine learning” approaches (classifiers) to combine information on the comparison between expression signal of the last 50 bp of the first gene (upstream) in the pair, of the intergenic region (up to 50 bp), of the first 50 bp of the second gene (downstream), and also on the length of the intergenic region. The rationale is that genes in a same operon are transcribed at the same level and are separated by a short intergenic region. Comparison between expression levels relies on statistics derived from the Kruskal-Wallis test. The classifiers are trained using operon information from the MicrobesOnline database. Performance are compared to two previously proposed methods (Rockhopper and DOOR) and a simple voting strategy is proposed to combine the results of the different classifiers.

As tackled in this work, the problem of operon prediction is fairly simple excepted for reasons stemming from the notion of operon which does not reflect the full complexity of bacterial transcriptomes (transcription units can overlap and transcription termination be partial). In this context, the proposed methods appears as ad-hoc solutions that are simple and work (even if maybe not well theoretically grounded, see major comments below). It is not clear that they outperform previously proposed approaches. For these reasons, my general feeling is that the manuscript is of limited originality and interest.

**Response: We thank the reviewer for his/her time in providing this review. We have addressed the specific concerns outlined below. For the reviewer’s more general criticisms, we would be happy to address these if the reviewer could provide more specific or constructive information on how the manuscript could be improved.**

Major comments:

* The introduction states the problem of operon prediction without mentioning the fact that the notion of pairs of genes in operon or not cannot describe the full complexity of bacterial transcriptomes. My personal opinion is that the problem tackled in this work is only of limited interest.

**Response: We agree that bacterial transcriptomes are more complex than just operon prediction. However, in this work, we are just addressing the problem of operon prediction, and are not aiming to address the full complexity of the bacterial transcriptome. In response to this comment, we have however included a feature in OperonSEQer that threads together operons based on pairwise predictions, and have mentioned this in the text.**

* The introduction does not provide enough insights on the principles underlying the different operon predictions methods already described in the literature over the years (from co-transcription, to intergenic distance and prediction of intrinsic terminators...).

**Response: We have added additional information about operon prediction to the introduction (highlighted).**

* The text seems often superficial or trivial.

**Response: We have addressed all the individual issues in the text that were brought up by reviewers to improve the flow and clarity of the manuscript. It would help to have some specificity on exactly where the reviewer encounters sections that were superficial, so that can be addressed.**

* L201-202 I do not understand the use of the subsampling approach to establish confidence intervals (a bootstrap would be more appropriate). I did not find more details in the M&M section.

**Response: We have used a bootstrap approach and clarified this in the methods section.**

* L204-211 I do not understand the interest of these comments about the difference in recall vs. accuracy trade-offs between the different classifiers when this trade-off can simply be tuned by changing the cut-off on the probability of the prediction (as done to draw the ROC curves in Sup Figure 2). The AUC is probably the appropriate measure to compare the classifiers in this context.

**Response: We appreciate the reviewer’s comment that each individual model can be tuned to tradeoff between recall and specificity. However, the tradeoff made by any single model is limited by the biases of that model when it comes to how it calls the operon. In other words, due to biases, there may be some operons that are never called by any one model, regardless of how much it is tuned. Therefore, we feel like having multiple models removes any individual model bias (in terms of the recall-specificity axis) but still allows for the flexibility described by the reviewer. We have clarified this in the text. The individual AUC values remain in the manuscript as a method to compare the individual classifiers.**

* L220-231 The authors show that by combining the results of the different classifiers with a simple voting scheme they can modify the recall vs. accuracy trade-off but they do not demonstrate that this allow to outperform (in terms of AUC) the best of their classifiers. In this context, the statement made L307-308 does not seem rigorously supported by the results presented.

**Response: We have now included a comparison of the AUC scores of the individual classifiers and the ensemble for the test data sets.**

* As presented I do not fully grasp the point analysis of SMRT-seq operon pairs. The authors seem to put forward the idea that their method is interesting because it can make true predictions (i.e. validated by some data sets) that are not in the standard lists of operons. This is not really surprising and is probably expected for any reasonable de novo method given the complexity of the bacterial transcriptome.

**###Response: We have replaced Figure 4A with a AUC analysis (as previously suggested by the reviewer for other figures) and have clarified the conclusion of this figure in the main text. In a nutshell, we aim to show that this method can detect novel operons without requiring the newest RNA-sequencing technology (as is the case in SMRT-seq), but can take advantage of deposited data to extract additional information.**

* The material and methods is not described with enough details. For instance it is not clear how paired-end reads are handled in the bioinformatic workflow and if the distribution of counts that are compared between regions are the numbers of reads starting at each position or the numbers of reads overlapping each position within each of the three regions (with which option genomecov is used?). Importantly in the second case (overlapping reads or fragments) the use of Kruskal-Wallis test does not seem theoretically justified since the length of the reads or fragments induces an important correlation between counts.

**Response: We have added increased clarity to the methods (specifically the addition of the description of the -pc option for paired-end reads in genomecov, which was accidentally omitted previously in the methods section). In addition, the point is well taken regarding the length of the reads affecting correlations – we have moved the window 50bp into the gene body to remove some of the correlation between counts due to read/fragment length. We understand that we cannot fully eliminate the possibility of reads/fragments that overlap two genes and an intergenic region, but with the gaps we are using, the probability is significantly lower. As per the data we have collected, the mean length of an intergenic region is 180bp. By shifting the region we read into the gene body by 50 bp on either end, a fragment would have to be 380bp to span the entire region, which would be very rare in most cases (fragments typically range from 200 – 400 bp without adapters).**

* Given the typical short length of the intergenic region and the typical fragment length (nowadays around 300 bp for pair-end “short-read” datasets?) I do not fully understand why the authors does not simply examine whether there are reads that overlap the two adjacent genes in order to see if they are co-transcribed.

**Response: The idea behind this approach is that it wouldn’t require reads spanning junctions between genes, but that it would be amenable to it if that were to happen at a subset of genes. Since intergenic regions vary significantly in size, requiring junction-spanning reads across three reads will have different probabilities of occurring, thereby confounding the analysis. Thus, using an approach that uses signal distribution rather than read distribution is more generalizable method. In addition, while it is true that for genome sequencing applications, reads of up to 300bp are often used, for the vast majority of RNA-sequencing applications, the longest recommended read length by (for example) Illumina is 2 x 75bp (**[**https://www.illumina.com/science/technology/next-generation-sequencing/plan-experiments/read-length.html**](https://www.illumina.com/science/technology/next-generation-sequencing/plan-experiments/read-length.html)**).**

Some minor comments:

* Spelling of the name of the proposed program is not always consistent (OperonSEQer or Operon-SEQer)

**Response: We have edited all instances to the non-hyphenated form.**

* The text contains typos (“an technological opportinity” L79, “resoureces” L138, ...)

**Response: We have reviewed the manuscript and corrected all typos.**

* L92 it is a mistake to oppose “non-parametric” and “normal”. Many parametric statistical methods have been developed to analyze data from distributions that are not normal.

**Response: We have adjusted the text to not oppose non-parametric and normal.**

* L225 does the authors mean Supp Table 1 instead of Supp Figure 3?

**Response: Yes, we have corrected this reference in the ‘ChangesTracked’ version of the manuscript.**

* L276-277 analysis of single condition should not be presented as the opposed of a comparison between two experimental conditions. Many data sets contains much more than two experimental conditions...

**Response: We have adjusted this to reflect that we are comparing single to multiple experimental conditions**
